# Severe Late-Onset Anthracycline-Induced Cardiotoxicity in Breast Cancer Survivor Patient: Importance of Post-Chemotherapy Surveillance

**DOI:** 10.1016/j.jaccas.2024.103189

**Published:** 2025-03-19

**Authors:** Rizky Hendiperdana, Amiliana M. Soesanto

**Affiliations:** a Division of Cardiovascular Medicine. Pandan Arang General Hospital, Boyolali, Central Java, Indonesia; b Department Cardiology and Vascular Medicine, Faculty of Medicine University of Indonesia/National Cardiovascular Centre Harapan Kita, Jakarta, Indonesia

**Keywords:** cardiotoxicity, chemotherapy, echocardiography, longitudinal strain, myocardial work

## Abstract

**Background:**

Left ventricular (LV) dysfunction is a common and serious complication from cancer treatment. One of the common regimens that can cause cardiotoxicity is anthracycline, and anthracycline-induced cardiotoxicity may manifest after several years.

**Case Summary:**

A 32-year-old woman presented with heart failure syndrome late after chemotherapy administration for breast cancer therapy. Several months after chemotherapy completion, patient presented with overt LV dysfunction. The guideline-directed medical treatment was initiated. Patient demonstrated gradual recovery and progressive improvement of myocardial systolic performance with LV thrombus resolution after 5-month evaluation.

**Discussion:**

The recognition of cancer therapy–related cardiac dysfunction (CTRCD) is important since early detection and treatment with heart failure treatment provide a good functional recovery and long-term prognosis. The clinical application of longitudinal strain and myocardial work echocardiography in CTRCD cases for therapeutic response tracking and prognostication purposes is recommended.

**Take-Home Message:**

Long-term postchemotherapy echocardiography evaluation especially in high-risk CTRCD criteria is of utmost importance due to the possibility of late-onset CTRCD.

## Graphical Abstract

**Figure:**
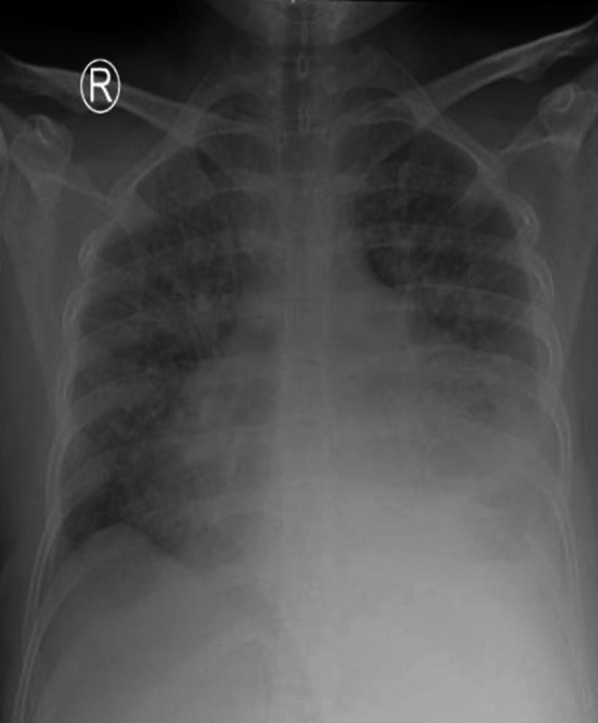

**Visual Summary:**
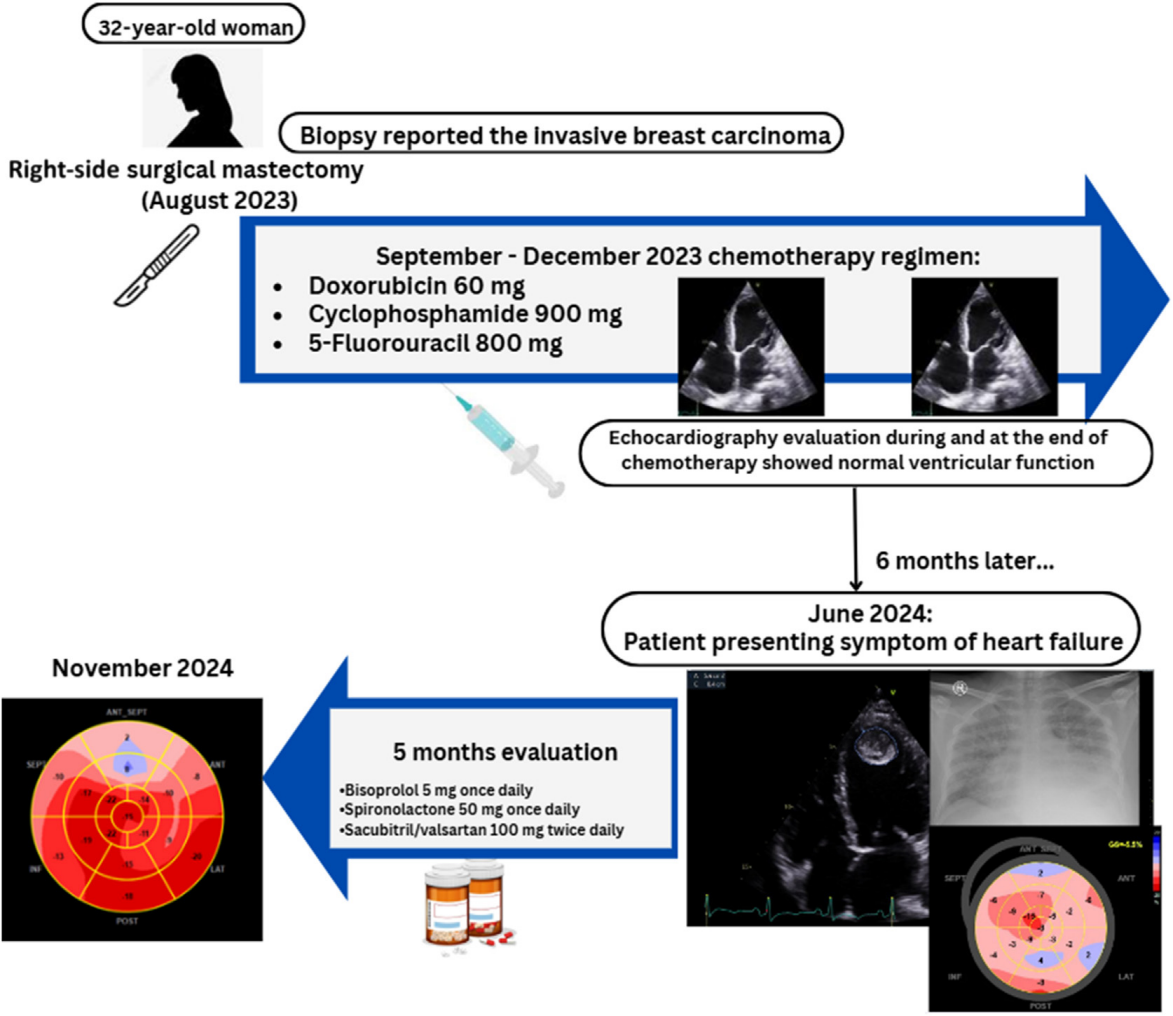
Clinical Timeline

## History of Presentation

A 32-year-old woman presented to the cardiology outpatient service (June 2024) with exertional dyspnea, fatiguing easily, with chest pain for the previous month. Patient noticed increased chest pain intensity for 2 weeks. Patient has a history of breast cancer with right-sided mastectomy with a chemotherapy protocol. Patient denied a history of hypertension, diabetes mellitus, or recent pregnancy.

**Take-Home Messages**

• This case highlights the importance of long-term post-chemotherapy echocardiography evaluation considering the possibility of late-onset CTRCD, especially in patients meeting high-risk CTRCD criteria.
• This case emphasizes the clinical application of longitudinal strain and myocardial work echocardiography in CTRCD cases for therapeutic response tracking and prognostication purposes as well.

Vital signs showed blood pressure of 88/57 mm Hg, heart rate 64 beats/min and 96% peripheral oxygen saturation, and body surface area of 1.75 m^2^. Physical examination was unremarkable. Basic initial transthoracic echocardiography (TTE) showed severely reduced left ventricular (LV) ejection fraction (LVEF) of 23% with thrombus at the LV apex.

## Past Medical History

The patient has a history of right-side surgical mastectomy for breast cancer in the previous 9 months before patient presentation (August 2023). The biopsy reported the invasive breast carcinoma of NST grade 2 of 3 with tumor infiltrating lymphocytes 20%. The immunochemistry examination result was as follows: estrogen receptor (ER) positive, progesterone receptor (PR) positive, Her2: negative (score +1) with Ki67: proliferative index 50%. The profile is suitable for luminal B molecular subtype with negative Her2.

After surgical management, the patient was administered 5 cycles of chemotherapy with a regimen that consisted of cyclophosphamide 900 mg, doxorubicin 60 mg, and 5-fluorouracil (5-FU) 800 mg once a month for 4 months’ duration (September 2023 until December 2023). In the middle of the chemotherapy cycle, echocardiography evaluation was performed in November 2023 and December 2023 with the result of a normal echocardiogram without any structural heart abnormality (LVEF 58%). The chemotherapeutic agent was discontinued in December 2023. The subsequent management adjuvant therapy was tamoxifen 10 mg orally and goserelin 3.6 mg subcutaneously once a month. The regiment continued until the patient presented with symptoms at our center.

## Differential Diagnosis

The patient presentation clearly suggested heart failure with reduced ejection fraction (HFrEF). The differential diagnosis of possible etiologies was first, related to cancer therapy-related cardiac dysfunction (CTRCD) due to the recent history of chemotherapy administration. The medical history of normal echocardiograms during chemotherapy sessions suggest the possibility of late-onset CTRCD. Second, the differential diagnosis was coronary artery disease etiology.

## Investigation

Blood test examination results showed normal cardiac troponin I of 0.03 ng/mL, with subclinical hyperthyroid (TSH = 0.131 mIU/L and FT4 = 1.8 ng/dL) and normal serum creatinine = 0.8 mg/dL; other laboratory results were unremarkable.

**Figure 1:**
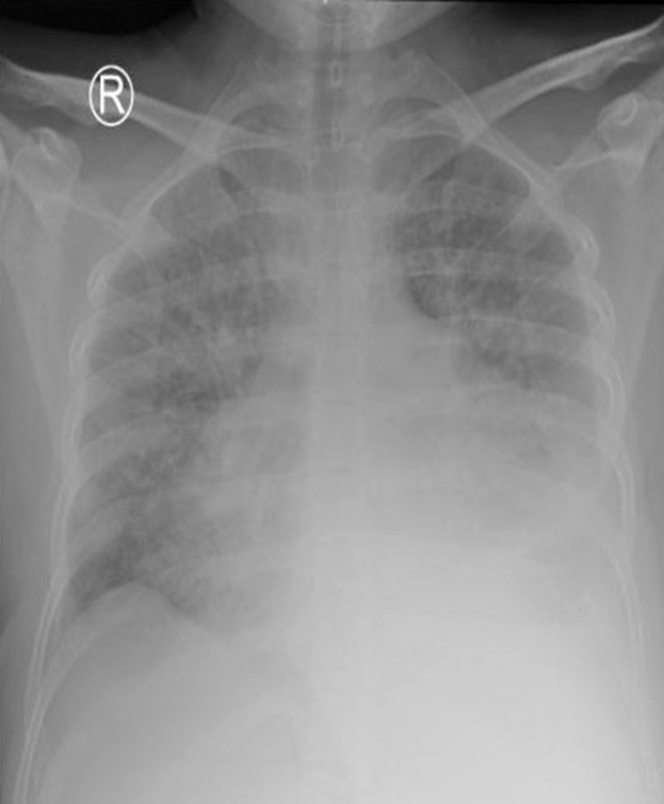
Chest X-Ray at Initial Presentation Chest x-ray at initial presentation showed cardiomegaly with increased pulmonary vascular markings.

**Figure 2:**
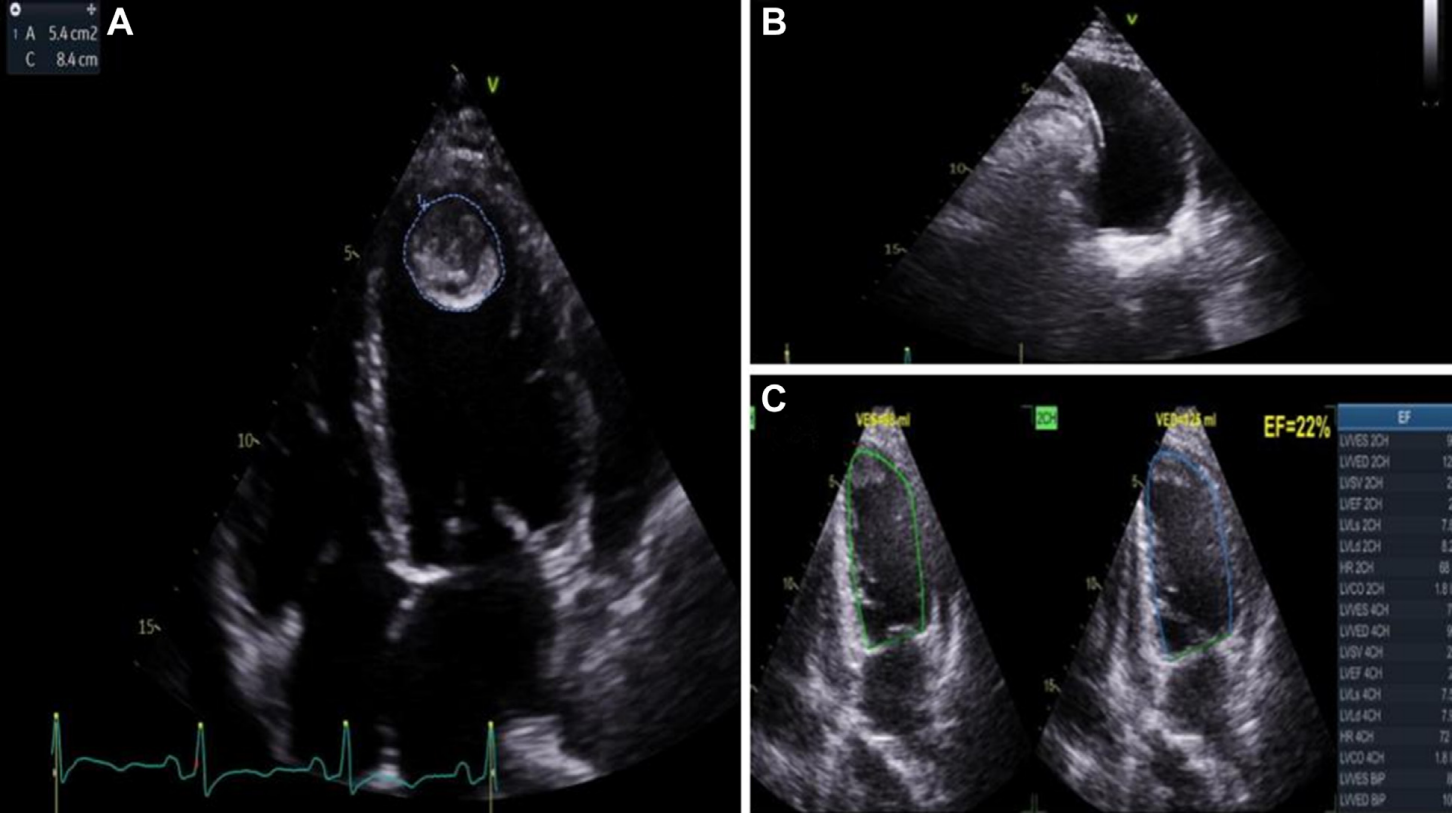
Baseline Echocardiographic Assessment at Initial Presentation (A) The 4-chamber view showed large apical left ventricular (LV) thrombus; (B) lung ultrasound revealed right-sided pleural effusion; (C) Simpson method ejection fraction (EF) measurement showed EF of 22%.

Chest roentgen showed cardiomegaly with increased pulmonary vascular marking (Figure 1). TTE showed the following: LVEF 23% (Simpson) with left atrial and LV dilatation, mild mitral regurgitation, mild reduced right ventricular systolic function (TAPSE [tricuspid annular plane systolic excursion] 15 mm, RV S′ 8 cm/s) with 5.4 cm^2^ thrombus at the LV apex. Pleural effusion was also noted (Figure 2, Video 1).

**Figure 3:**
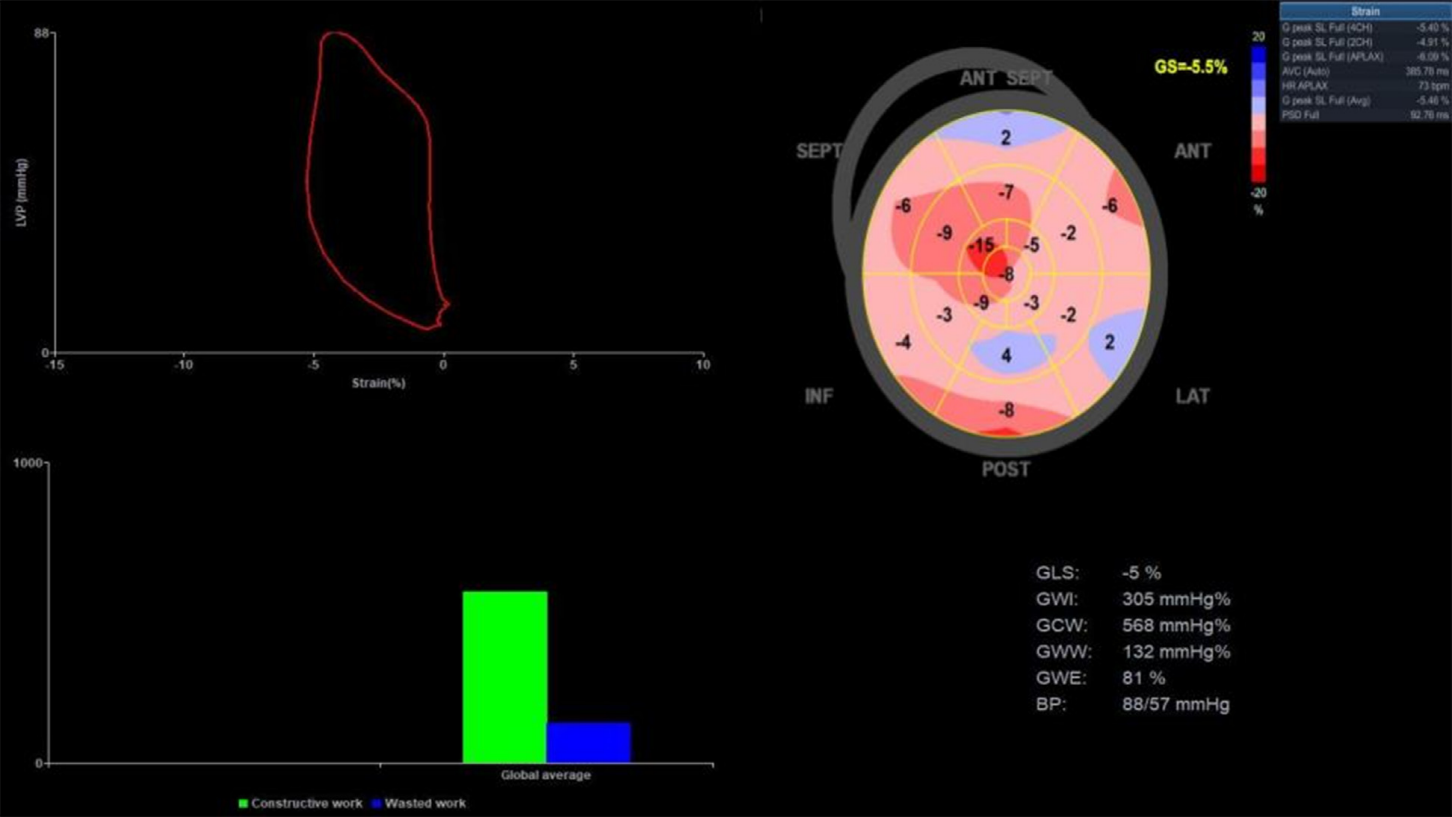
Baseline GLS and MWIs at Initial Presentation ANT = anterior; BP = blood pressure; GCW = global constructive work; GLS = global longitudinal strain; GS = global strain; GWE = global work efficiency; GWI = global work index; GWW = global wasted work; INF = inferior; LAT = lateral; POST = posterior; SEPT = septal.

**Figure 4:**
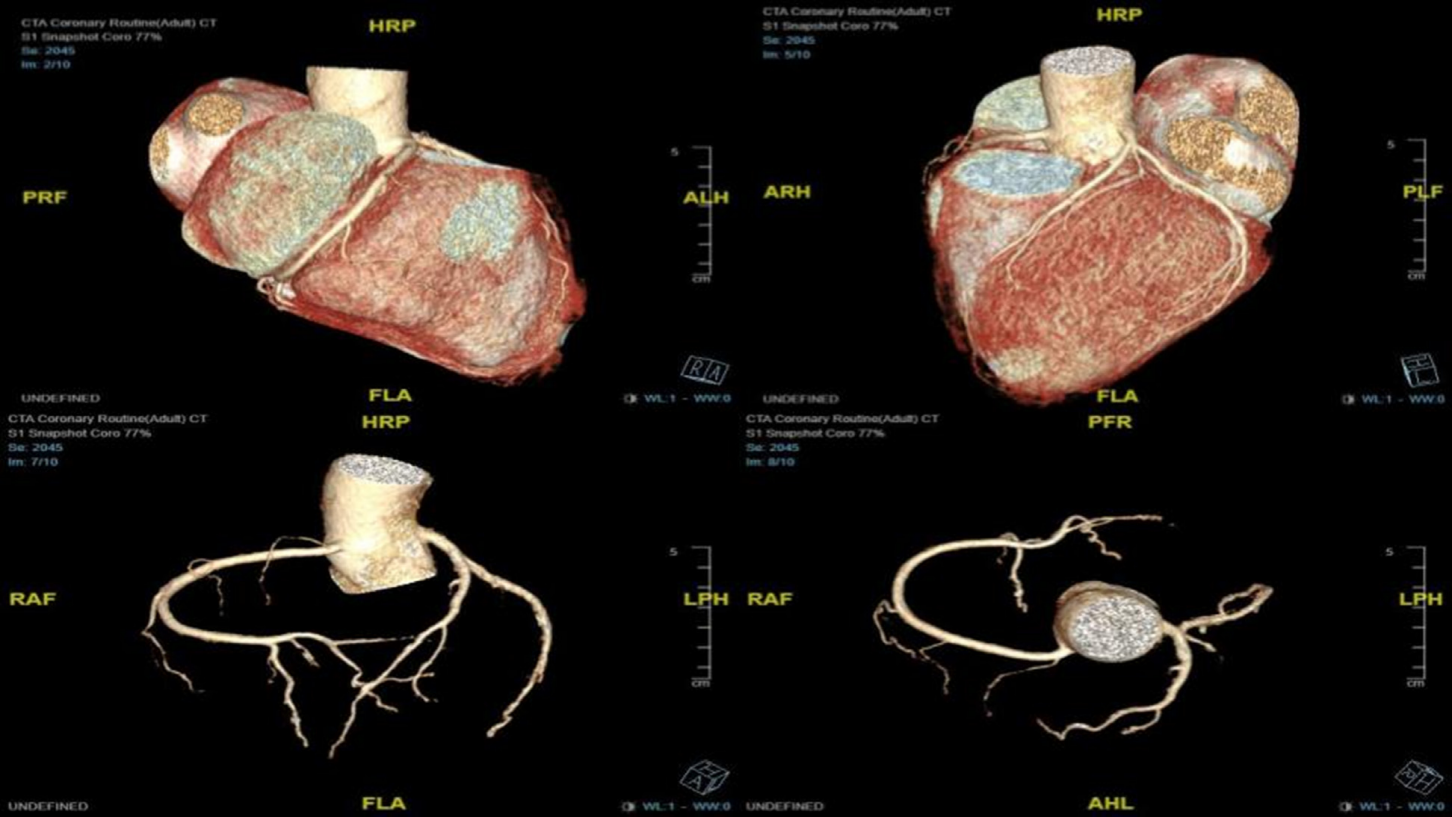
Coronary Computed Tomography Angiography Revealed Normal Coronary Anatomy AHL = anterior head left; ALH = anterior left head; ARH = anterior right head; FLA = feet left anterior; HRP = head right posterior; LPH = left posterior head; PRF = posterior right feet; PLF = posterior left feet; RAF = right anterior feet.

Further echocardiography modality was performed to assess speckle tracking–derived LV strain with average LV global longitudinal strain (GLS) of −5.3%. Pressure-strain loop–derived myocardial work index (MWI) showed global work index (GWI) of 305 mm Hg%, global constructive work (GCW) of 568 mm Hg%, and global work efficiency (GWE) of 81% (Figure 3). Coronary computed tomography angiography showed normal coronary anatomy without stenosis with a zero calcium score (Figure 4).

## Management

The diagnosis of late-onset CTRCD due to the anthracycline, cyclophosphamide, and 5-FU regimen has been made. Management was planned according to the American College of Cardiology and European Society of Cardiology guidelines for HFrEF management.^1^^,^^2^ Patient was administered ramipril uptitrated to 10 mg daily, bisoprolol 10 mg daily, spironolactone up to 50 mg daily, and atorvastatin 40 mg daily for the cardioprotective effect with oral diuretic furosemide therapy for congestion symptoms.

## Outcome and Follow-Up

After the 2-month evaluation (August 2024), the dyspnea symptom was resolved with minimal persistent chest pain. TTE evaluation showed an LVEF of 27%; although the LVEF measurement showed no improvement in systolic function, GLS and myocardial work showed significant improvement trajectory. The GLS and MWI results are as follows: GLS −10%, GWI 839 mm Hg%, GCW 1,044 mm Hg%, global wasted work 112 mm Hg%, and GWE 88%.

**Figure 5:**
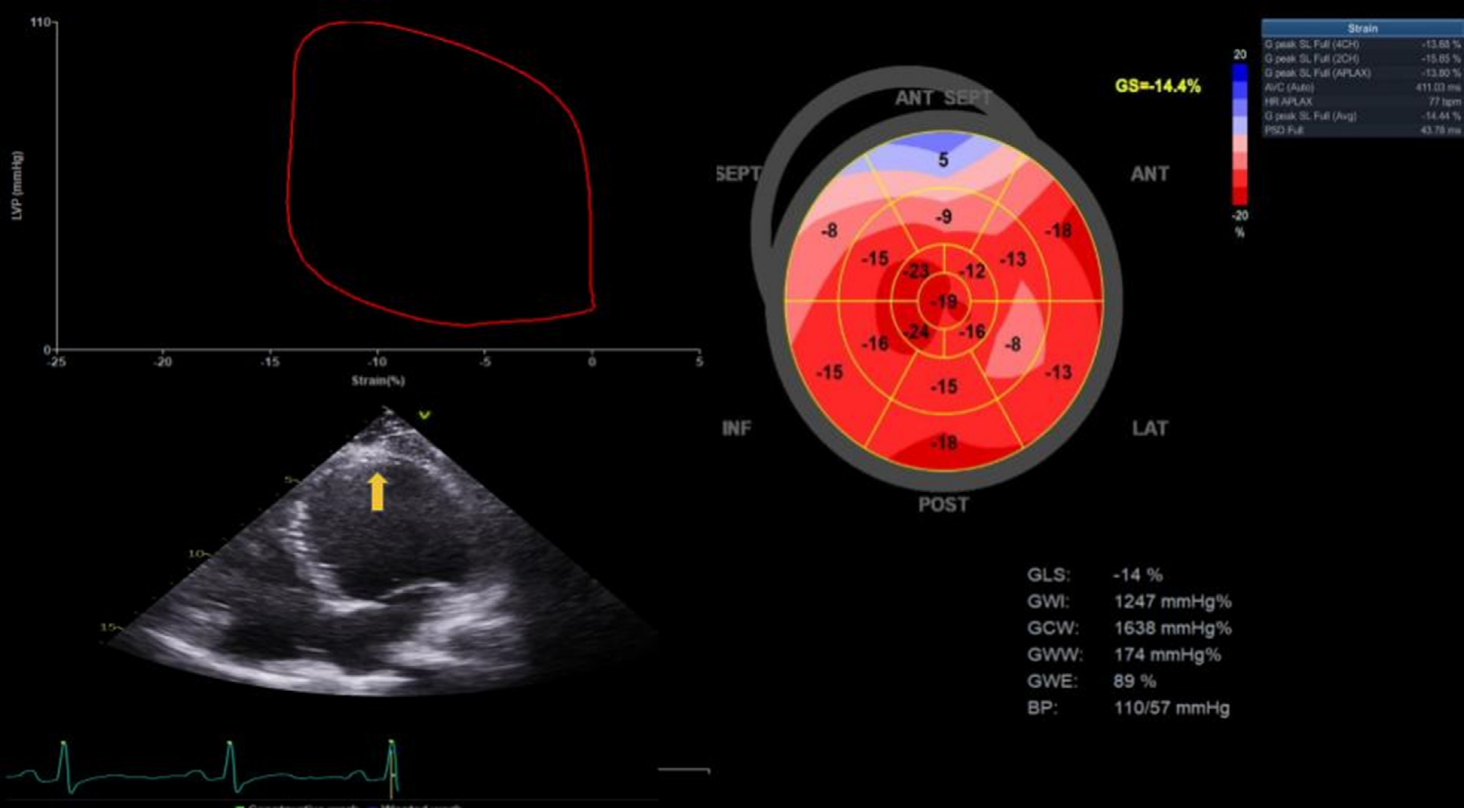
GLS and MWIs After 5-Month Treatment Lower left panel shows the 4-chamber view with resolution of LV thrombus (yellow arrow). Abbreviations as in Figures 2, 3, and 4.

Given the insight of initial recovery, we replaced ramipril with sacubitril/valsartan 100 mg twice daily for the subsequent management regiment. Continuing monitoring after 5 months (November 2024) showed further myocardial recovery. The LVEF significantly improved to 47% with resolution of LV thrombus. This improvement was also followed by an increase in GLS of −14.4% and GWI of 1,247 mm Hg%, GCW of 1,638 mm Hg%, global wasted work of 174 mm Hg%, and GWE of 89% (Figure 5, Video 2). The patient's chest pain gradually resolved by the last outpatient visit. The patient has returned to normal daily routine activity uneventfully.

## Discussion

We report a case of a 32-year-old woman with severe CTRCD based on the echocardiographic finding after chemotherapy regimen completion. LV dysfunction with HFrEF consequences is a common and serious complication from cancer treatment.^3^ From a chronological point of view, the possibility of late-developing CTRCD is strongly suggested due to documented normal ventricular function by echocardiographic evaluation at the end of the chemotherapy regimen as recommended.^4^ The patient was administered the anthracycline-based drug doxorubicin, cyclophosphamide regimen, and 5-FU. While the patient was administered below the cumulative dose for cardiac toxicity for each regimen, CTRCD developed later after completion of this chemotherapy regimen. The cumulative dose for cardiac toxicity for doxorubicin, cyclophosphamide, and 5-FU are 550 mg/m^2^, 1,500 mg/m^2^/d, and 2,800 mg/m^2^, respectively.^5^

Each chemotherapy regimen has its own specific mechanism of inducing cardiotoxicity with various cardiac toxicity incidences. Cyclophosphamide is a DNA-alkylating agent with high incidence of cardiac toxicity (around 20%-40%), followed by 5-FU (around 25%), and the least incidence is doxorubicin (about 7%-25%). The risk of CTRCD is increased in the cumulative manner of each risk. The common pathobiology mechanism of these chemotherapy regimens is to cause cardiomyocyte inflammation and swelling and to induce apoptosis of cardiomyocytes. 5-FU is described to have an effect to induce myocardial ischemia.^5^ Hence, our recent case had a substantial increased risk for cardiac toxicity.

Our case’s echocardiographic evaluation during and after chemotherapy completion was performed showing good myocardial systolic function by LVEF measurement. However, the patient developed overt CTRCD in the following months after the last chemotherapy regimen. Anthracycline-induced cardiotoxicity (AIC) may manifest after several years (median of 7 years after treatment).^3^

There are several risk criteria for AIC occurrence. The cumulative dose, female sex, age >65 years or pediatric population <18 years, kidney failure, pre-existing cardiovascular comorbidity, and concomitant chemotherapeutic agent use, especially an alkylating agent such as cyclophosphamide, are the proposed criteria that will increase AIC.^3^ Our patient meets the high-risk criteria for AIC due to female sex and concomitant use of a cyclophosphamide regimen. It is proposed in a patient with 1 or more risk criteria that the cumulative dose vs cardiotoxicity curve is shifted to the left, and this warrants careful echocardiographic monitoring.^3^ The importance of clinician vigilance in the postchemotherapy period for echocardiographic evaluation and expanded monitoring window period is strongly suggested due the possibility of late-developing CTRCD, especially in multiple chemotherapy regimens.

The recognition CTRCD is important since early detection and treatment with heart failure treatment have a good functional recovery and long-term prognosis.^3^ We performed conventional LVEF measurement, GLS assessment, and MWI analysis for the purpose of detecting subtle changes in improvement or worsening cardiac function. In the first 2-month evaluation after heart failure therapy was administered, we found no significant improvement in LVEF value but clear improvement from GLS and MWI evaluation. It is well-described that these more sensitive modalities for evaluating systolic performance are recommended in the cardio-oncology field for the purpose of recognizing the subclinical functional alteration, to tracking the therapeutic response or for prognostication purposes.^6^^,^^7^

Our case showed overt LV systolic dysfunction with a clear clinical indication for HFrEF therapy. In the HFrEF therapeutic course, patients demonstrated gradual myocardial recovery, which is shown by improvement in LVEF, GLS, and MWIs values. Real-world cohort data on CTRCD patient revealed more than one-half of patients with CTRCD achieved myocardial recovery by LVEF improvement.^8^ The combination of guideline-directed medical treatment for HFrEF and meticulous echocardiography monitoring has become the key process in managing symptomatic overt CTRCD.^1^, ^2^, ^3^, ^4^^,^^9^^,^^10^

In the low-risk population, echocardiography screening is recommended at the fourth cycle of anthracycline regimen (Class IIb) and 12 months post-treatment (Class I). Meanwhile, in the baseline high- and very high-risk AIC population, echocardiographic screening is recommended in second, fourth, and sixth cycle of corresponding anthracycline regimen (Class 1) but also 3 and 12 months after treatment (Class 1).^10^

High or very high risk for future AIC can extend to more than 30 years; it is important to note that AIC is a lifetime risk. Therefore, it is recommended that post-treatment echocardiogram surveillance is performed in 1, 3, and 5 years after chemotherapy completion, and every 5 years thereafter for a lifetime, unless symptoms occurs.^9^^,^^10^

## Conclusions

Cancer therapy–related cardiac dysfunction can have late manifestation after the end of the therapeutic cycle. Even if patients have normal cardiac function after chemotherapy, the clinical vigilance to expand the monitoring window is strongly suggested since the occurrence of CTRCD in the first year after chemotherapy completion is common. Expert consensus strongly suggested the use of more sensitive surrogate marker of myocardial systolic performance for detection and prognosticate CTRCD cases.

## Funding Support and Author Disclosures

The authors have reported that they have no relationships relevant to the contents of this paper to disclose.

## Appendix

Video 1 Baseline 4-chamber with apical LV thrombus

Video 2 Follow up 4-chamber view echocardiography
